# Knockdown of c-Myc expression by RNAi inhibits MCF-7 breast tumor cells growth *in vitro *and *in vivo*

**DOI:** 10.1186/bcr975

**Published:** 2004-12-17

**Authors:** Yi-hua Wang, Shuang Liu, Guo Zhang, Cui-qi Zhou, Hong-xia Zhu, Xiao-bo Zhou, Lan-ping Quan, Jin-feng Bai, Ning-zhi Xu

**Affiliations:** 1 Laboratory of Cell and Molecular Biology, Cancer Institute & Cancer Hospital, Chinese Academy of Medical Sciences and Peking Union Medical College, Beijing, PR China

**Keywords:** c-Myc, gene therapy, MCF-7, RNA interference

## Abstract

**Introduction:**

Breast cancer is the leading cause of cancer death in women worldwide. Elevated expression of c-Myc is a frequent genetic abnormality seen in this malignancy. For a better understanding of its role in maintaining the malignant phenotype, we used RNA interference (RNAi) directed against c-Myc in our study. RNAi provides a new, reliable method to investigate gene function and has the potential for gene therapy. The aim of the study was to examine the anti-tumor effects elicited by a decrease in the protein level of c-Myc by RNAi and its possible mechanism of effects in MCF-7 cells.

**Method:**

A plasmid-based polymerase III promoter system was used to deliver and express short interfering RNA (siRNA) targeting c-*myc *to reduce its expression in MCF-7 cells. Western blot analysis was used to measure the protein level of c-Myc. We assessed the effects of c-Myc silencing on tumor growth by a growth curve, by soft agar assay and by nude mice experiments *in vivo*. Standard fluorescence-activated cell sorter analysis and TdT-mediated dUTP nick end labelling assay were used to determine apoptosis of the cells.

**Results:**

Our data showed that plasmids expressing siRNA against c-*myc *markedly and durably reduced its expression in MCF-7 cells by up to 80%, decreased the growth rate of MCF-7 cells, inhibited colony formation in soft agar and significantly reduced tumor growth in nude mice. We also found that depletion of c-Myc in this manner promoted apoptosis of MCF-7 cells upon serum withdrawal.

**Conclusion:**

c-Myc has a pivotal function in the development of breast cancer. Our data show that decreasing the c-Myc protein level in MCF-7 cells by RNAi could significantly inhibit tumor growth both *in vitro *and *in vivo*, and imply the therapeutic potential of RNAi on the treatment of breast cancer by targeting overexpression oncogenes such as c-*myc*, and c-*myc *might be a potential therapeutic target for human breast cancer.

## Introduction

Breast cancer is the leading cause of cancer death in women worldwide. Despite advances in detection and chemotherapy, many women with breast cancer continue to die of this malignancy [1]. Therefore, an understanding of the molecular mechanisms involved in breast cancer formation and progression should be helpful in developing more effective treatments for breast cancer.

c-Myc is believed to participate in most aspects of cellular function, including replication, growth, metabolism, differentiation, and apoptosis [2]. Previous studies indicate that c-Myc activates a variety of known genes as part of a heterodimeric complex with Max [2]. A frequent genetic abnormality seen in breast cancer is the elevated expression of c-Myc [3,4]. The importance of c-Myc expression in breast cancer is demonstrated both by studies of transgenic mice and by clinical research [3,5]. Abnormal expression of c-*myc *transgenes in the mouse mammary gland is associated with an increased incidence of breast carcinomas [5]. Moreover, clinical studies have indicated that c-Myc is important in the development and progression of breast cancer, in that overexpression of c-Myc was found in most breast cancer patients and was correlated with poor prognosis in those patients [3].

The role of c-Myc in breast cancer has been extensively examined in many studies for the past decade [6]; however, specifically reducing its level by genetic means in established breast cancer cell lines is still helpful for a better understanding of its role in maintaining the malignant phenotype. Thus, in this study, we investigated whether specifically decreasing the protein level of c-Myc in a breast cancer cell line in which this protein was overexpressed might result in the inhibition of cell growth *in vitro *and *in vivo*. For this purpose, RNA interference (RNAi) directed against c-*myc *was used.

RNAi is the sequence-specific gene silencing induced by double-stranded RNA (dsRNA). This phenomenon is conserved in a variety of organisms: *Caenorhabditis elegans*, *Drosophila*, plants, and mammals. RNAi is mediated by short interfering RNAs (siRNAs) that are produced from long dsRNAs of exogenous or endogenous origin by an endonuclease of the ribonuclease-III type, called Dicer. The resulting siRNAs are about 21–23 nucleotides (nt) long and are then incorporated into a nuclease complex, the RNA-inducing silencing complex, which then targets and cleaves mRNA containing a sequence identical to that of the siRNA [7]. Rapid progress has been made in the use of RNAi [8]. More recently, a technical breakthrough came from the demonstration that dsRNA of 19–29 nt expressed endogenously with RNA polymerase III promoter induced target gene silencing in mammalian cells [9]. The expression of siRNA from DNA templates offers several advantages over chemically synthesized siRNA delivery. Hairpin siRNAs transcribed from a vector are thought to suppress the expression of targeted genes more efficiently, less expensively and more easily than synthesized siRNA [10].

Here we used a plasmid-based polymerase III promoter system to deliver and express siRNA targeting c-*myc *to determine whether this technique could be used for the specific inhibition of oncogene overexpression and whether this inhibition resulted in antitumor effects. We showed in our study that specific downregulation of c-Myc by RNAi was sufficient to inhibit the growth of MCF-7 cells *in vitro *and *in vivo*, and that c-*myc *might serve as a therapeutic target for human breast cancer.

## Method

### Plasmid construction

To generate c-Myc knockdown vector, one annealed set of oligonucleotides encoding short hairpin transcripts corresponding to nt 1906–1926 of c-*myc *mRNA (GenBank accession no. NM-002467) [11] were cloned into p*Silencer *1.0_U6 (Ambion; hereafter abbreviated to p*Silencer*). In brief, the short-hairpin-RNA-encoding complementary single-stranded oligonucleotides, which hybridized to give overhangs compatible with *Apa*I and *Eco*RI, were designed with a computer program available on the Internet . Oligonucleotides encoding short hairpin RNAs were then ligated into p*Silencer*. Bacterial colonies were pooled and used for plasmid preparation. The positive clones were confirmed by sequencing. The resulting plasmid was designated as p*Silencer*–c-Myc.

### Cell line and cell culture

The breast cancer adenocarcinoma cell line MCF-7 was obtained from the American Type Culture Collection (Manassas, VA). The cells were grown in Dulbecco's modified Eagle's medium (Invitrogen) supplemented with 10% fetal bovine serum (Gibco BRL), 50 units/ml penicillin, and 50 μg/ml streptomycin. The MCF-7 cells were maintained in a humidified 37°C incubator with 5% CO_2_, fed every 3 days with complete medium, and subcultured when confluence was reached.

### Transfection of cells

A total of 2 × 10^5 ^cells were seeded into each well of a six-well tissue culture plate (Costar). The next day (when the cells were 70–80% confluent), the culture medium was aspirated and the cell monolayer was washed with prewarmed sterile phosphate-buffered saline (PBS). Cells were transfected with the appropriate plasmids by using LipofectAMINE reagent (Invitrogen) in accordance with the manufacturer's protocol. The cells were harvested at different time points. Western blot analysis or other experiments were performed.

### Western blot analysis

Cells were harvested at different time points and lysed in mammalian cell lysis buffer, then western blot analysis was performed with the use of conventional protocols as described previously [12]. In brief, the protein concentration was determined with a bicinchoninic acid kit with bovine serum albumin as a standard (Pierce). Equal amounts of total protein were then separated on 12% polyacrylamide gels by using standard sodium dodecyl sulfate–polyacrylamide gel electrophoresis (SDS–PAGE) techniques, then transferred to nitrocellulose membranes (PROTRAN). The antibodies and dilutions used included anti-c-Myc (9E10; 1:1000 dilution; Santa Cruz) and anti-β-actin (AC-15; 1:5000 dilution; Sigma), and after being washed extensively the membranes were incubated with anti-mouse IgG–horseradish peroxidase conjugate antibody (Zhongshan Company) for 1 hour at room temperature and developed with a Luminol chemiluminescence detection kit (Santa Cruz). Membranes probed for c-Myc were reprobed for β-actin to normalize for loading and/or quantification errors and to allow comparisons of target protein expression to be made. Protein expression was quantified with a Gel EDAS analysis system (Cold Spring USA Corporation) and Gel-Pro Analyzer 3.1 software (Media Cybernetics).

### Cell growth assay

At 2 days after transfection, MCF-7 cells transfected with indicated plasmids were harvested and replated at a density of 50 cells/mm^2 ^in triplicate. The total cell number was quantified every 2 days with a hematocytometer and an Olympus inverted microscope. Cell viability was assessed by using trypan blue.

### Soft agar colony assay

At 2 days after transfection, MCF-7 cells (300 cells per well) transfected with indicated plasmids were mixed with tissue culture medium containing 0.7% agar to result in a final agar concentration of 0.35%. Then 1 ml samples of this cell suspension were immediately plated in six-well plates coated with 0.6% agar in tissue culture medium (2 ml per well) and cultured at 37°C with 5% CO_2_. After 2 weeks the top layer of the culture was stained with 0.2% *p*-iodonitrotetrazolium violet (Sigma). The culture was analyzed in triplicate, and colonies larger than 100 μm in diameter were counted.

### Tumor growth in nude mice

Equal numbers (10^6 ^or 2 × 10^6^) of MCF-7 cells transfected with p*Silencer*–c-Myc or p*Silencer *were harvested by trypsinization 2 days after transfection, washed twice with 1 × PBS, and resuspended in 0.2 ml of saline. Two groups of five 4–6-week-old female nude mice were then given bilateral subcutaneous injections with control cells or cells transfected with plasmids against c-Myc. The mice were kept in pathogen-free environments and checked every 2 days. The date at which a palpable tumor first arose and the weight of the tumor were recorded.

### Cell cycle analysis

Standard fluorescence-activated cell sorting analysis was used to determine apoptosis of the cells. In brief, MCF-7 cells were transfected with p*Silencer*–c-Myc or p*Silencer*; 24 hours later, cells were deprived of serum for 36 hours. Then cells were harvested, washed once in PBS and stained with propidium iodide (BD Biosciences). The apoptotic cells were assessed by flow cytometric detection of sub-G1 DNA content.

### TdT-mediated dUTP nick end labelling assay

Apoptotic cells were confirmed with the *in situ *cell death detection kit, Alkaline Phosphatase (Roche Applied Science), in accordance with the manufacturer's instructions. In brief, MCF-7 cells were grown on coverslips. The next day, cells were transfected with p*Silencer*–c-Myc or p*Silencer*. At 24 hours after transfection, cells were deprived of serum for 36 hours. Coverslips with adherent cells were fixed in 4% paraformaldehyde for 1 hour at room temperature and permeabilized with 0.1% Triton X-100 for 2 min on ice. DNA fragments were labeled with the TdT-mediated dUTP nick end labelling (TUNEL) reaction mixture for 60 min at 37°C in a humidified atmosphere in the dark. The coverslips were then incubated with Converter alkaline phosphatase for 30 min at 37°C in a humidified chamber, rinsed in PBS, and incubated with nitro blue tetrazolium/5-bromo-4-chloroindol-3-yl phosphate (Roche Applied Science) for 10 min. Cells were mounted cell side downward on a microscope slide, and the apoptotic cells (dark blue staining) were counted under a microscope. Three fields were randomly counted for each sample.

### Statistical analysis

SPSS for Windows (SPSS Inc.) was used to analyze the data and plot curves. A two-tailed unpaired *t*-test was used to compare the statistical significance of the differences in data from the two groups.

## Results

### Suppression of c-Myc overexpression in MCF-7 cells by RNAi

**Figure 1:**
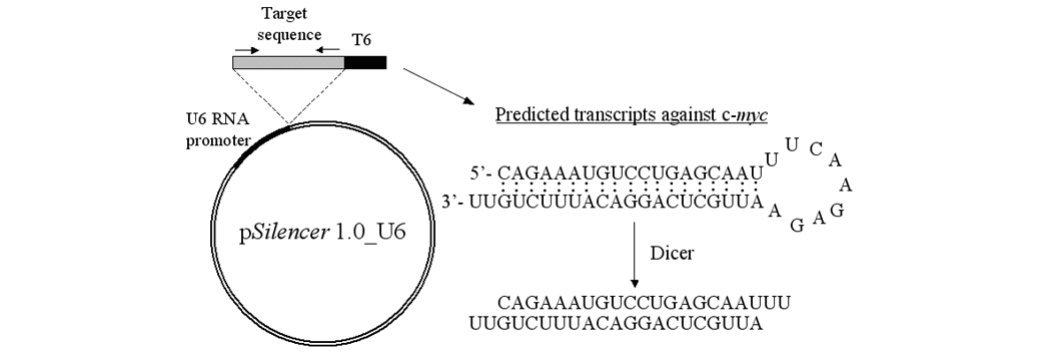
Schematic drawing of the p*Silencer*1.0_U6 vector. The U6-RNA promoter was cloned in front of the gene-specific targeting sequence (19-nucleotide sequences from the target transcript separated by a short spacer from the reverse complement of the same sequence) and six thymidines (T_6_) as a termination signal. The predicted secondary structure of the p*Silencer*–c-Myc transcript target c-Myc is shown. The transcript, a short hairpin double-stranded RNA (dsRNA), is believed to be further cleaved by Dicer to generate a 21-nucleotide siRNA that forms dsRNA–endonuclease complexes and will bind and destroy c-*myc *mRNA.

**Figure 2:**
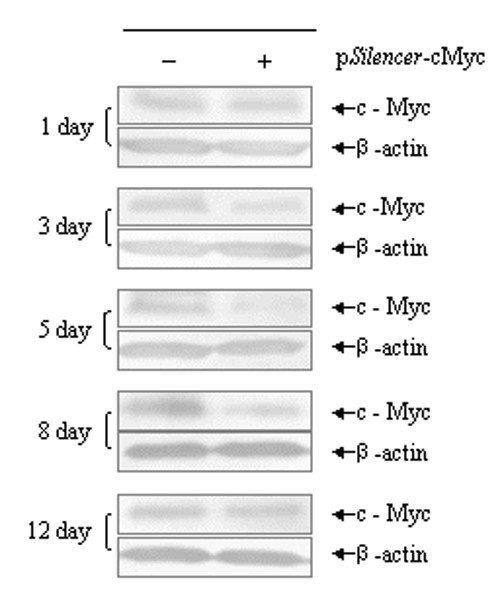
Time course of the reduction in c-Myc protein levels by p*Silencer*–c-Myc. Exponentially proliferating MCF-7 cells were transfected with p*Silencer*–c-Myc or p*Silencer *and whole cell lysates were prepared at the time points indicated. Total cell lysates were separated by SDS–polyacrylamide-gel electrophoresis and immunoblotted with an antibody against c-Myc; expression levels were normalized for loading by probing for β-actin.

We designed and synthesized one pair of oligonucleotides encoding short hairpin transcripts directed against a portion of the c-*myc *mRNA. This pair of oligonucleotides was then ligated into p*Silencer*. The plasmid of p*Silencer*–c-Myc contains a U6 promoter that directs the synthesis of oligonucleotides in an inverted repeat with 9 nt for its loop, with six T bases added at the end to serve as a termination signal for RNA polymerase III. The RNA is expected to fold back to form a hairpin loop structure after being transcribed; the hairpin dsRNA can then be further cleaved by Dicer to generate a 21-nucleotide siRNA, the active form for the RNAi effect, which will form dsRNA–endonuclease complexes and will bind and destroy c-*myc *mRNA inside cells [8] (Fig. 1).

p*Silencer*–c-Myc was transfected into MCF-7 cells and its effects on c-Myc protein levels were determined by comparison with p*Silencer*-transfected cells by western blot at the time points indicated. We found that the c-Myc expression levels were suppressed by up to 80% in MCF-7 cells at day 5 after transfection. Actually the levels of c-Myc were decreased as early as 24 hours after transfection and remained at low levels until 12 days after transfection (Fig. 2). The inhibitory effect was shown to be specific because transfection with p*Silencer *did not alter c-Myc levels. In addition, RNAi did not cause a nonspecific downregulation of gene expression, as determined by the β-actin control (Fig. 2). Similar results with c-Myc RNAi were also seen in other cell lines including HCT116 and HepG2 (data not shown). These data indicated that vector-based RNAi could effectively suppress c-Myc overexpression and resulted in prolonged decreases in specific cellular gene expression without marked effects on other cellular proteins.

### Decreased levels of c-Myc significantly alter the growth rate of MCF-7 cells

**Figure 3:**
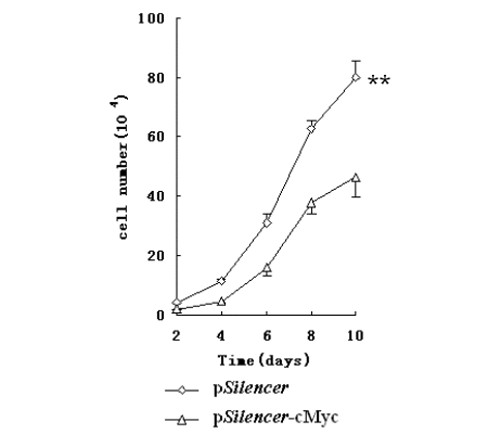
RNAi directed against c-Myc leads to a reduced cellular growth rate. MCF-7 cells were transfected with p*Silencer*–c-Myc or p*Silencer*. After 48 hours the cells were trypsinized and replated at a density of 50 cells/mm^2 ^in triplicate. Cells were counted every 2 days. The data shown are means and SD from three independent experiments. **P < 0.01.

Previous studies have shown that c-Myc is important in cellular proliferation and cell growth [6]. Thus, increased levels of c-Myc might have a function in the growth advantage seen in breast tumors. In our study, MCF-7 cells were transfected with p*Silencer*–c-Myc or p*Silencer*. The number of MCF-7 cells was then counted every 2 days after transfection. Our data showed that RNAi directed against c-*myc *significantly decreased the growth rate of MCF-7 cells, with a 50–60% decrease at different time points repeatedly in three separate experiments (Fig. 3).

### Decreases in c-Myc protein inhibit colony formation

**Figure 4:**
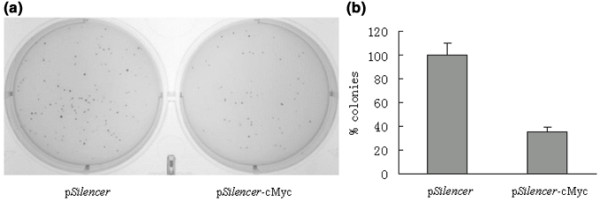
Knockdown of c-Myc by RNAi reduces colony formation in soft agar. MCF-7 cells were transfected with p*Silencer*–c-Myc or p*Silencer *as controls, and seeded in 0.35% agarose containing Dulbecco's modified Eagle's medium with 10% fetal bovine serum. The colony numbers were counted 2 weeks later. **(a) **Representative wells demonstrating the total number of colonies formed by MCF-7 transfected with the indicated plasmids. **(b) **The numbers of colonies of p*Silencer*–c-Myc-treated cells standardized against the control cells (set at 100%). The data shown are means and SD from two independent triplicate experiments. The difference between treatments is statistically significant (*P *< 0.001).

We then tested whether RNAi-mediated reductions in c-Myc levels could influence the ability of MCF-7 cells to form colonies in soft agar. MCF-7 cells were transfected with p*Silencer*–c-Myc or p*Silencer*. At 48 hours after transfection, the cells were placed into medium with soft agar, and colonies were counted after 2 weeks. RNAi directed against c-*myc *resulted in a significant decrease (about 65%) in colony formation in MCF-7 cells (Fig. 4). The smaller number of colonies in the p*Silencer*–c-Myc group than in the control group was statistically significant (*P *< 0.001). These results showed that the reduction in c-Myc protein level decreased the ability of breast cancer cells to form colonies in soft agar.

### RNAi directed against c-Myc reduces tumor growth in nude mice

To address the potential effects of RNAi *in vivo *on inhibiting the growth of breast cancer cells, equal numbers (10^6 ^or 2 × 10^6^) of MCF-7 cells transfected with p*Silencer*–c-Myc or p*Silencer *were injected into female nude mice (five animals for each treatment). At 5 or 8 weeks after injection of these cells, the mice were killed and the weights of the tumors were recorded. As seen in Table 1, when 10^6 ^cells were injected into nude mice, tumors were seen 8 weeks later in three of five at the left lateral where MCF-7 cells transfected with p*Silencer *were injected, whereas none were seen at the right lateral where MCF-7 cells transfected with p*Silencer*–c-Myc were injected. In addition, 5 weeks after 2 × 10^6 ^cells were injected into nude mice, tumors were seen in four of five at the left lateral where MCF-7 cells transfected with p*Silencer *were injected, whereas none were seen at the right lateral where MCF-7 cells transfected with p*Silencer*–c-Myc were injected (Table 1). Thus, c-Myc RNAi significantly suppressed tumor growth in nude mice in comparison with control, indicating that targeting c-*myc *by RNAi could exert a strong antitumor effect *in vivo *on MCF-7 cells.

### Induction of apoptosis in MCF-7 cells by RNAi depletion of c-Myc upon serum deprivation

**Figure 5:**
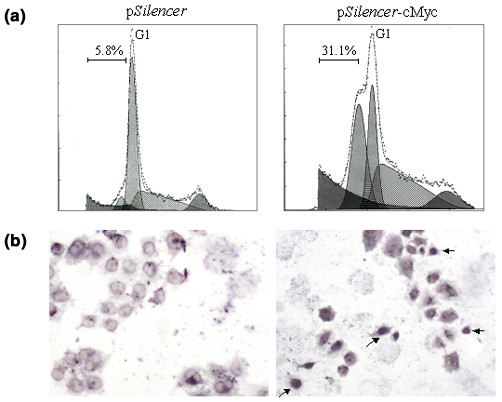
Apoptosis induced after serum deprivation by depletion of c-Myc in MCF-7 cells. **(a) **Downregulation of c-Myc promoted apoptosis of MCF-7 cells after serum deprivation. MCF-7 cells were transfected with p*Silencer*–c-Myc or p*Silencer*. After 24 hours, cells were deprived of serum for 36 hours. Cells were then collected by trypsinization. The apoptotic cells were determined by flow cytometry. The cell population in sub-G1 is shown. The *x *and *y *axes show DNA content and cell number, respectively. **(b) **TUNEL assay to detect apoptotic cells *in situ*. MCF-7 cells were grown on coverslips and transfected with p*Silencer*–c-Myc or p*Silencer*. After 24 hours, cells were deprived of serum for 36 hours. Cells were then analyzed for apoptosis with the TUNEL assay. Dark-blue staining of nuclei indicates apoptosis. Arrows indicate representative TUNEL-positive cells.

The above data demonstrated that knockdown of c-Myc in MCF-7 cells could significantly inhibit the growth of tumor cells both *in vitro *and *in vivo*. To determine whether depletion of c-Myc could promote the death of tumor cells, flow cytometry and TUNEL assays were performed. At 24 hours after transfection with p*Silencer*–c-Myc or p*Silencer*, MCF-7 cells were deprived of serum for 36 hours. These cells were then analyzed by flow cytometry or TUNEL assay. Significant sub-G1 (apoptotic) populations were observed in the flow cytometry assay. We found that 31.1% of MCF-7 cells transfected with p*Silencer*–c-Myc underwent apoptosis after serum starvation, compared with 5.8% in the control group (Fig. 5a). We also confirmed the apoptosis of MCF-7 cells by TUNEL assay (Fig. 5b). About 40% cells were TUNEL-positive in the p*Silencer*–c-Myc group, compared with 6% in the control group (*P *< 0.01). These data suggested that depletion of c-Myc by RNAi in MCF-7 cells made the cells more sensitive to apoptosis after serum deprivation.

## Discussion

Cancer cells often show alteration in the signal-transduction pathways, leading to proliferation in response to external signals. Oncogene overexpression is a common phenomenon in the development and progression of many human cancers. Oncogenes therefore provide a potential target for cancer gene therapy [13].

The important oncogene c-*myc *is expressed in a high proportion of most human cancers, including breast, prostate, gastrointestinal cancer, lymphoma, melanoma, and myeloid leukemia [14]. In its physiological role, c-Myc is broadly expressed during embryogenesis and in tissue compartments of the adult that possess high proliferative capacity. Altered expression of c-Myc seems to define a common event associated with the pathogenesis of most human cancers [6]. Previous studies demonstrated that the continued presence of c-Myc was required for cancer development and not just for initiation, and inactivation of c-Myc resulted in the sustained regression of tumors [15-17]. Similar results were also observed in breast cancer. D'Cruz and colleagues demonstrated that overexpression of c-Myc by an inducible system in the mammary epithelium of transgenic mice resulted in the formation of invasive mammary adenocarcinomas, many of which regressed fully after c-Myc deinduction [18].

Therefore, specific downregulation of c-Myc might be a potential therapeutic strategy against human cancers, including breast cancer. In fact, the antagonists of c-Myc, including full-length antisense mRNA [19], oligonucleotides against c-*myc *mRNA [20] or a dominant-negative mutant [21], were previously reported to inhibit proliferation of cancer cell lines *in vitro*. However, it was only successful in some situations; these technologies have been difficult to apply universally [22]. Recently the advent of RNAi-directed 'knock-down' has sparked a revolution in somatic cell genetics, allowing the inexpensive, rapid analysis of gene function in mammals, and might be exploited for gene therapy [7,8]. Some studies directly compared RNAi with antisense RNA and found that RNAi seemed to be quantitatively more efficient and durable in cell culture and in nude mice [23].

By means of the RNAi method, in the present study, cellular growth assays, both *in vitro *and *in vivo*, were used to determine the functional consequences of RNAi-mediated decreases in of c-Myc in established breast cancer cells. Our results demonstrated that RNAi can effectively downregulate oncogene overexpression with great specificity. We showed that the plasmids endogenously expressing siRNA could successfully deplete up to 80% of c-Myc expression in MCF-7 cells at day 5 after transfection. Furthermore, the tumor inhibition effects persisted for at least 12 days after transfection in dishes and for 2 months in nude mice as shown by experiments *in vitro *and *in vivo*, even though the protein level of c-Myc in silenced clones expressing siRNA was back to almost the same level as in the control cells by day 12 after transfection. Our data were consistent with the results of Jain and colleagues [17].

They showed that within 24 hours of c-*myc *inactivation, the osteogenic sarcoma cells flattened and showed less cell division. And even after reactivation of c-*myc *expression in these cultured cells, total cell numbers continued to be lower. Less than 1% of the tumor cells regained their neoplastic growth properties [17]. All of these data indicated that brief inactivation of c-Myc could induce a sustained loss of neoplastic phenotypes. Moreover, other groups using chemically synthesized siRNAs to knock down their favored oncogenes also found that a transient decrease in oncogene expression could inhibit the growth of tumor cells *in vitro *and/or *in vivo *[8]. Nevertheless, the underlying mechanism of this phenomenon in MCF-7 cells should be further investigated.

Although some studies previously revealed that the effects of inactivation of c-Myc in some cell lines were modest [6], other groups using different approaches to reduce the protein level of c-Myc found that a decrease in c-Myc expression could inhibit the growth of these tumor cells, including breast tumor cells [19-21]. Nevertheless there were still conflicting results on whether c-Myc expression was necessary to maintain tumorigenesis in different animal models from different laboratories [15-18,24,25]. For example, some studies showed that the role of oncogenic c-Myc in tumor maintenance was essential and that all effects of c-Myc *in vivo *were reversible, in that without continuous c-Myc activation there would even be regression of established tumors back to phenotypically normal in transgenic mouse models [15,16,24]. Similar phenomena were observed by other groups focusing on other oncogenes, such as *bcr*/*abl *[26] and *H*-*ras *[27]. However, there were also conflicting reports, mainly showing that brief inactivation of c-Myc could induce sustained loss of neoplastic phenotypes in certain animal models [17,18,25]. It was notable that without secondary oncogenic mutation, spontaneously or selectively, such as *Kras2 *oncogene mutation, in nearly all breast tumors induced by conditionally expressing the human c-Myc in the mammary epithelium of a transgenic mouse model, deinduction of c-Myc protein could lead to full regression of tumors [18]. Similar data were also obtained by Karlsson and colleagues that the inactivation of c-Myc alone was found to be sufficient to cause sustained tumor regression in c-Myc-induced hematopoietic tumors; in contrast, tumor cells that acquired novel chromosomal translocations relapsed independently of Myc to maintain their neoplastic phenotype [25]. It was therefore not surprising for us to show here that a transient reduction of c-Myc protein level by RNAi could significantly inhibit the growth rate of MCF-7 cells and its ability to form colonies in soft agar. Additionally, the remarkable effect in nude mice supported the effectiveness of this treatment.

Our data also suggested that knockdown of c-Myc by RNAi in MCF-7 cells could increase the sensitivity of these cells to apoptotic stimuli, such as serum starvation. This was most probably one of the reasons for the anti-tumor effects. There have previously been conflicting reports about the role of c-Myc in apoptosis [3]. Constant overexpression of c-Myc might induce apoptosis [2,6], and a decrease in c-Myc levels by techniques brought about by, for example, an antisense approach might also cause apoptosis of certain tumor cells [28-30] or might increase the sensitivity of the cells to apoptotic stimuli [31]. These conflicting observations suggested that c-Myc was capable of both inducing and suppressing apoptosis in different types of tumor cell, under different conditions, and in different systems. In MCF-7 cells, suppression of c-Myc expression in response to aromatase inhibitors or topoisomerase α inhibitors could induce these cells to apoptosis [32,33]. However, the pathways that c-Myc controls and/or that are involved in the observed apoptosis remain obscure. D'Agnano and colleagues suggested that in melanoma cells the downregulation of c-Myc by an antisense approach could activate apoptosis by increasing the levels of p27^Kip1 ^[28]. Overexpression of the cyclin-dependent kinase inhibitor p27^Kip1 ^was able to promote apoptosis in several mammalian tumor cell lines [34]. However, understanding the precise pathway by which a decrease in c-Myc in MCF-7 cells by RNAi was able to induce apoptosis upon serum deprivation needs further study.

So far, RNAi has been used to inhibit virus-induced diseases (for example HIV [35] and influenza [36]), oncogenic *K-ras*, *H-ras*-induced tumorigenesis [37,38], activation of oncogenes resulting from chromosomal translocations (for example *bcr*/*abl *in chromic myeloid leukemia [39]), cancers caused by viral infections [40], and so on. Recently, retroviral-based approaches to deliver siRNA into tissue-cultured mammalian cells have been proved to be powerful [37,38], and doxycycline-regulated inducible knockdown of gene expression by RNAi has been shown to be particularly useful for the analysis of genes that are essential for cellular survival [41,42]. These studies have marked a new era in the genetic manipulation of human cancer development by allowing oncogenes to be downregulated by RNAi.

## Conclusion

In summary, RNAi has been used in this study to demonstrate that decreases in c-Myc levels can inhibit tumor growth in assays both *in vivo *and *in vitro*. In addition, these data indicate that RNAi provides a useful method with which to study the role of genes that control the growth of cancer cells; and, given its specificity and the lower doses needed to inhibit gene expression than those required for antisense oligonucleotides, RNAi might have potential therapeutic utility in a variety of disease states, including cancers. Future studies could further test whether c-Myc overexpression can be efficiently depleted by siRNA expressed from a DNA-based expression vector combined with a tumor-specific promoter, such that RNAi can specifically target oncogenes in cancer cells without affecting normal cells.

## Abbreviations

dsRNA = double-stranded RNA; nt = nucleotides; PBS = phosphate-buffered saline; RNAi = RNA interference; SDS–PAGE = sodium dodecyl sulfate–polyacrylamide gel electrophoresis; siRNA = short interfering RNA.

## Competing interests

The author(s) declare that they have no competing interests.

## Authors' contributions

YW designed the experiments, constructed the plasmids, performed transfections, and wrote the manuscript. SL constructed the growth curve and performed the nude mice experiments. GZ performed the western blots and the soft agar assay. CZ, HZ, and XZ performed the flow cytometry and TUNEL experiments. LQ and JB conducted the cell culturing. NX is the corresponding author. All authors read and approved the final manuscript.

## Figures and Tables

**Table 1:** RNAi directed against c-Myc significantly inhibits MCF-7 cell growth in nude mice

No. of cells^a^	p*Silencer*				p*Silencer*–c-Myc			
	
	Tumor weight (mg)	Tumors	Injections	Latency^b ^(day)	Tumor weight (mg)	Tumors	Injections	Latency^b ^(day)
10^6^	45–210	3	5	42–49	0	0	5	>56
2 × 10^6^	145–2248	4	5	21–28	0	0	5	>35

^a^Number of cells transfected with the indicated plasmids that were injected subcutaneously, bilaterally into five nude mice.

^b^The time interval required for a palpable tumor to arise.

## References

[B1] WHO (2003). World Health Report 2003.

[B2] Dang CV (1999). c-Myc target genes involved in cell growth, apoptosis, and metabolism. Mol Cell Biol.

[B3] Liao DJ, Dickson RB (2000). c-Myc in breast cancer. Endocr Relat Cancer.

[B4] Doisneau-Sixou SF, Sergio CM, Carroll JS, Hui R, Musgrove EA, Sutherland RL (2003). Estrogen and antiestrogen regulation of cell cycle progression in breast cancer cells. Endocr Relat Cancer.

[B5] Hutchinson JN, Muller WJ (2000). Transgenic mouse models of human breast cancer. Oncogene.

[B6] Pelengaris S, Khan M, Evan G (2002). c-Myc: more than just a matter of life and death. Nat Rev Cancer.

[B7] Hannon GJ (2002). RNA interference. Nature.

[B8] Dykxhoorn DM, Novina CD, Sharp PA (2003). Killing the messenger: short RNAs that silence gene expression. Nat Rev Mol Cell Biol.

[B9] Sui G, Soohoo C, Affar EB, Gay F, Shi Y, Forrester WC, Shi Y (2002). A DNA vector-based RNAi technology to suppress gene expression in mammalian cells. Proc Natl Acad Sci USA.

[B10] Yu JY, DeRuiter SL, Turner DL (2002). RNA interference by expression of short-interfering RNAs and hairpin RNAs in mammalian cells. Proc Natl Acad Sci USA.

[B11] Williams NS, Gaynor RB, Scoggin S, Verma U, Gokaslan T, Simmang C, Fleming J, Tavana D, Frenkel E, Becerra C (2003). Identification and validation of genes involved in the pathogenesis of colorectal cancer using cDNA microarrays and RNA interference. Clin Cancer Res.

[B12] Zhou CQ, Liu S, Xue LY, Wang YH, Zhu HX, Lu N, Xu NZ (2003). Down-regulation of gamma-synuclein in human esophageal squamous cell carcinoma. World J Gastroenterol.

[B13] Malumbres M, Barbacid M (2001). To cycle or not to cycle: a critical decision in cancer. Nat Rev Cancer.

[B14] Nesbit CE, Tersak JM, Prochownik EV (1999). MYC oncogenes and human neoplastic disease. Oncogene.

[B15] Felsher DW, Bishop JM (1999). Reversible tumorigenesis by *MYC *in hematopoietic lineages. Mol Cell.

[B16] Pelengaris S, Littlewood T, Khan M, Elia G, Evan G (1999). Reversible activation of c-Myc in skin: induction of a complex neoplastic phenotype by a single oncogenic lesion. Mol Cell.

[B17] Jain M, Arvanitis C, Chu K, Dewey W, Leonhardt E, Trinh M, Sundberg CD, Bishop JM, Felsher DW (2002). Sustained loss of a neoplastic phenotype by brief inactivation of *MYC*. Science.

[B18] D'Cruz CM, Gunther EJ, Boxer RB, Hartman JL, Sintasath L, Moody SE, Cox JD, Ha SI, Belka GK, Golant A (2001). c-MYC induces mammary tumorigenesis by means of a preferred pathway involving spontaneous Kras2 mutations. Nat Med.

[B19] Steiner MS, Anthony CT, Lu Y, Holt JT (1998). Antisense c-*myc *retroviral vector suppresses established human prostate cancer. Hum Gene Ther.

[B20] Watson PH, Pon RT, Shiu RPC (1991). Inhibition of c-*myc *expression by phosphorothioate antisense oligonucleotide identifies a critical role for c-*myc *in the growth of human breast cancer. Cancer Res.

[B21] Sawyers CL, Callahan W, Witte ON (1992). Dominant negative *MYC *blocks transformation by *ABL *oncogenes. Cell.

[B22] Braasch DA, Corey DR (2002). Novel antisense and peptide nucleic acid strategies for controlling gene expression. Biochemistry.

[B23] Bertrand JR, Pottier M, Vekris A, Opolon P, Maksimenko A, Malvy C (2002). Comparison of antisense oligonucleotides and siRNAs in cell culture and *in vivo*. Biochem Biophys Res Commun.

[B24] Pelengaris S, Khan M, Evan GI (2002). Suppression of Myc-induced apoptosis in beta cells exposes multiple oncogenic properties of Myc and triggers carcinogenic progression. Cell.

[B25] Karlsson A, Giuriato S, Tang F, Fung-Weier J, Levan G, Felsher DW (2003). Genomically complex lymphomas undergo sustained tumor regression upon MYC inactivation unless they acquire novel chromosomal translocations. Blood.

[B26] Huettner CS, Zhang P, Van Etten RA, Tenen DG (2000). Reversibility of acute B-cell leukaemia induced by BCR-ABL1. Nat Genet.

[B27] Chin L, Tam A, Pomerantz J, Wong M, Holash J, Bardeesy N, Shen Q, O'Hagan R, Pantginis J, Zhou H (1999). Essential role for oncogenic Ras in tumor maintenance. Nature.

[B28] D'Agnano I, Valentini A, Fornari C, Bucci B, Starace G, Felsani A, Citro G (2001). Myc down-regulation induces apoptosis in M14 melanoma cells by increasing p27^Kip1 ^levels. Oncogene.

[B29] Leonetti C, D'Agnano I, Lozupone F, Valentini A, Geiser T, Zon G, Calabretta B, Citro G, Zupi G (1996). Antitumor effect of c-myc antisense phosphorothioate oligodeoxynucleotides on human melanoma cells in vitro and and in mice. J Natl Cancer Inst.

[B30] Balaji KC, Koul H, Mitra S, Maramag C, Reddy P, Menon M, Malhotra RK, Laxmanan S (1997). Antiproliferative effects of c-myc antisense oligonucleotide in prostate cancer cells: a novel therapy in prostate cancer. Urology.

[B31] Citro G, D'Agnano I, Leonetti C, Perini R, Bucci B, Zon G, Calabretta B, Zupi G (1998). c-myc antisense oligodeoxynucleotides enhance the efficacy of cisplatin in melanoma chemotherapy *in vitro* and in nude mice. Cancer Res.

[B32] Thiantanawat A, Long BJ, Brodie AM (2003). Signaling pathways of apoptosis activated by aromatase inhibitors and antiestrogens. Cancer Res.

[B33] Magnet KJ, Orr MS, Cleveland JL, Rodriguez-Galindo C, Yang H, Yang C, Di YM, Jain PT, Gewirtz DA (2001). Suppression of c-myc expression and c-Myc function in response to sustained DNA damage in MCF-7 breast tumor cells. Biochem Pharmacol.

[B34] Lloyd RV, Erickson LA, Jin L, Kulig E, Qian X, Cheville JC, Scheithauer BW (1999). p27^kip1^: a multifunctional cyclin-dependent kinase inhibitor with prognostic significance in human cancers. Am J Pathol.

[B35] Kitabwalla M, Ruprecht RM (2002). RNA interference – a new weapon against HIV and beyond. N Engl J Med.

[B36] Ge Q, McManus MT, Nguyen T, Shen CH, Sharp PA, Eisen HN, Chen J (2003). RNA interference of influenza virus production by directly targeting mRNA for degradation and indirectly inhibiting all viral RNA transcription. Proc Natl Acad Sci USA.

[B37] Brummelkamp TR, Bernards R, Agami R (2002). Stable suppression of tumorigenicity by virus-mediated RNA interference. Cancer Cell.

[B38] Yang G, Thompson JA, Fang B, Liu J (2003). Silencing of H-*ras *gene expression by retrovirus-mediated siRNA decreases transformation efficiency and tumor growth in a model of human ovarian cancer. Oncogene.

[B39] Scherr M, Battmer K, Winkler T, Heidenreich O, Ganser A, Eder M (2003). Specific inhibition of *bcr-abl *gene expression by small interfering RNA. Blood.

[B40] Milner J (2003). RNA interference for treating cancers caused by viral infection. Expert Opin Biol Ther.

[B41] Matsukura S, Jones PA, Takai D (2003). Establishment of conditional vectors for hairpin siRNA knockdowns. Nucleic Acids Res.

[B42] van de Wetering M, Oving I, Muncan V, Pon Fong MT, Brantjes H, van Leenen D, Holstege FC, Brummelkamp TR, Agami R, Clevers H (2003). Specific inhibition of gene expression using a stably integrated, inducible small-interfering-RNA vector. EMBO Rep.

